# Production of MLM-Type structured lipids from fish oil catalyzed by *Thermomyces lanuginosus *lipase

**DOI:** 10.1186/1753-6561-8-S4-P44

**Published:** 2014-10-01

**Authors:** Joab Sousa, Arthur Souza, Alexandre Torres, Denise Freire

**Affiliations:** 1 Universidade Federal do Rio de Janeiro, UFRJ, Rio de Janeiro, Brazil

## Background

Lipases are powerful tools for the syntheses of structured lipids (SL) which are triacylglycerols (TAG) having particular fatty acid residues at specific positions. The production of TAG with medium chain fatty acids (MCFA), in positions *sn-*1 and *sn*-3, and a long-chain fatty acid (LCFA), in the internal position, has recently increased due to its nutritional interest in applications as reduced calorie fats [1].

Molecular structure of TAGs influences the fatty metabolism in organisms. Consequently, it is possible to control and improve the nutritional and pharmaceutical properties of TAGs with a correct design of SL chemical structure. The dietary reference intake of long-chain n-3 PUFA is based on current intake of a healthy population, and consists of 135-270 mg a day of EPA+DHA [2]. The intake of reduced levels of long-chain n-3 fatty acids have negative consequences in human health, and might occur relatively frequently in situations of irregular fish consumption.

So, the aim this work is to produce food oil with a low calorific value, with high content of eicosapentaenoic (EPA) and docosapentaenoic (DHA) acids from fish oil, which is useful for people who suffer from obesity or metabolic disorders caused by lack of such polyunsaturated fatty acids (PUFA) in the metabolism.

## Methods

### Acidolysis reaction

A fixed amount of *Thermomyces lanuginosus *immobilized (Lipozyme TL IM) and free lipase (Lipozyme TL 100L) (2% wt% of total substrates) was used for the acidolysis reactions that were performed at 40 °C, in solvent-free media, at a molar ratio 1:2 (fish oil:free fatty acid) in thermostated-capped cylindrical glass vessels under magnetic stirring, for 24 h.

### Analysis of products

The product mixture was separated by thin-layer chromatography (TLC) on silica gel plates and developed with n-hexane/ethyl ether/acetic acid (70:30:1). After this procedure, TLC plates were air-dried and sprayed with 0.2% (w/v) 2,7-dichlorofluorescein in 95% ethanol and the bands were visualized by ultraviolet light. The various groups of compounds (triacylglycerols, free fatty acids, diacylglycerols and monoacylglycerols) were identified by comparison with standards. The bands corresponding to TAG were scraped from TLC plates methylated and analyzed [3].

## Results

It was possible to obtain TAG of MLM type by acidolysis of fish oil with caprylic acid. Higher incorporations of caprylic acid (50%) into the TAG in these conditions were attained with both forms of lipase. The oils generated in this process provide about 88 to 92mg of the mixture of EPA+DHA per g oil using free (TL 100L) and immobilized (TL IM) forms of *Thermomyces lanuginosus *lipase. This amount was adequate for daily intake of EPA+DHA besides generating oil with low calorie.

## Conclusions

The both forms tested of *Thermomyces lanuginosus *lipase was able to catalyze the incorporation of caprylic acid into fish oil.

The oil generated is process could be used as a dietary supplement for specific clinical situations, once it has been possible to produce of nutritionally valued oils, rich in EPA and DHA with low calorie.
